# *aptf-1* mutants are primarily defective in head movement quiescence during *C. elegans* sleep

**DOI:** 10.17912/micropub.biology.000148

**Published:** 2019-08-19

**Authors:** Bryan Robinson, Desiree L Goetting, Janine Cisneros Desir, Cheryl Van Buskirk

**Affiliations:** 1 Department of Biology, California State University Northridge, Northridge CA USA 91330

**Figure 1.**
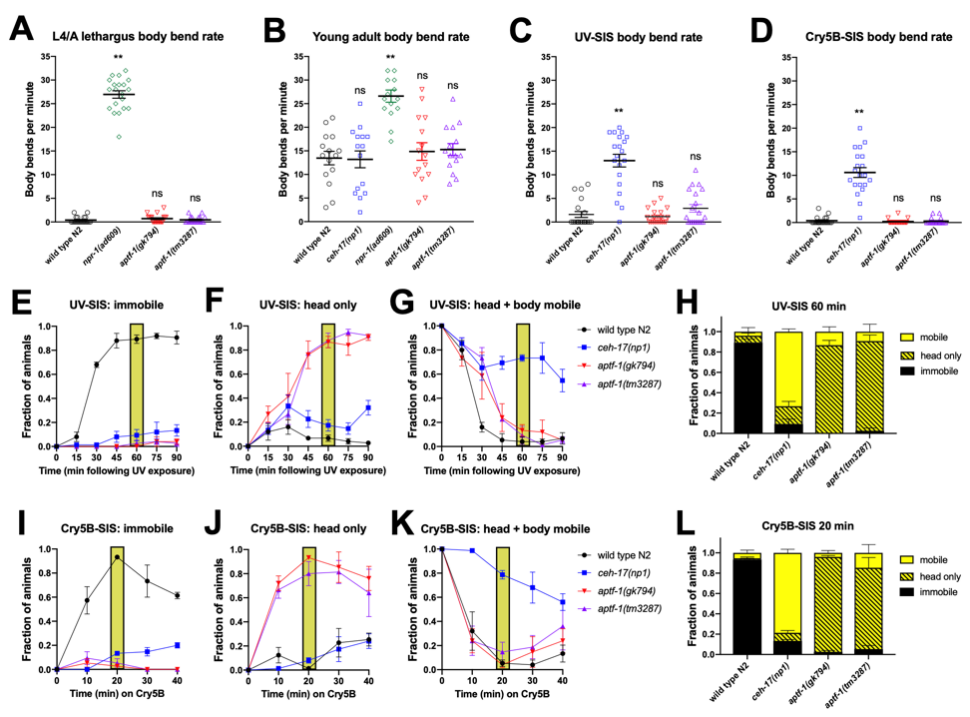
**Figure 1** **The RIS interneuron promotes head movement quiescence but not locomotor quiescence during lethargus and stress-induced sleep.** (A-D) Locomotor rate assessed by body bends per minute among animals during the L4-to-adult lethargus (A), during the young adult stage (B), and during stress-induced sleep at the young adult stage following exposure to ultraviolet light (C) or Cry5B pore-forming toxin (D). Animals were examined on thin even lawns of *E. coli* OP50 and examined for the number of sinusoidal body bends (peak-to-peak movement of any body part) in one minute. To select mid-lethargus animals, late-L4 larvae were identified based on vulval morphology, screened one hour later for cessation of pharyngeal pumping as an indicator of lethargus entry, and scored 30 min later for bend rates. For UV-SIS, worm plates were placed lid-down on a 302nm 60mW UV light box for 50 sec and examined 60 min later. For Cry5B-SIS, animals were exposed to Cry5B-expressing bacteria for 5 min, transferred to a thin even lawn of OP50, and examined 10-15 min later. For all bend rate analyses, plates were gently moved to the stereomicroscope field of view and allowed to sit for 1 min prior to examination. Each animal was examined for 1 min and a body bend was defined as a peak-to-peak unit of sinusoidal motion. **P<0.0001 vs. N2, one-way ANOVA with Dunnett’s multiple comparisons test. N=15-20, experimenter blind to genotype. (E-L) Categorization of movement during SIS following UV exposure (E-H) and during exposure to Cry5B (I-L). Each animal was examined for 5 sec and characterized as completely immobile (E and I), head mobile but body immobile (F and J), or head mobile plus body movement of at least 1/10 body-length (G and K). Two-way repeated measures (RM) ANOVA with time as the repeated factor and Sidak’s multiple comparisons test reveals that more *ceh-17(lf)* than *aptf-1(lf)* animals are completely immobile under certain SIS conditions (Panel I, *ceh-17* vs. each *aptf-1* mutant, P<0.05 at 40 min). However, *aptf-1* mutants are impaired for head movement quiescence (N2 vs. *aptf-1* mutants P<0.05 at multiple time points during UV-SIS and Cry5B-SIS), and not significantly impaired for body movement with the exception of the 30 min time point of UV-SIS, for which *aptf-1(tm3287)* is different from wild type N2 (P<0.05). H and L show stacked categories of behavior for the time points indicated by shaded boxes within the time courses. Three trials of 25 animals were performed and the experimenter was blind to genotype.

## Corrections

Corrections to this article were made. These corrections were recorded in an Erratum published on Aug 23, 2019.

## Description

Behavioral quiescence during sleep states can be measured by a variety of methods, each with advantages and limitations (Nagy et al., 2014). Centroid tracking gives information about locomotion (place-to-place movement) but may not register small head movements or other changes in body posture that do not change centroid position. Frame subtraction methods, based on analysis of pixel intensity changes between successive frames, register any movement as non-quiescent, but do not categorize the nature of movement. Counting body bends is a close proxy to locomotion (Karbowski et al., 2006) but can be time-consuming if done manually. Last, while nose tip movement has been observed to approximate body movement during *C. elegans* sleep (Bringmann, 2011), this approximation may not apply to mutants defective in only one of these behaviors. Here we provide an example of such a case.

*C.* *elegans* undergoes robust developmentally-timed sleep (DTS), also known as lethargus, prior to each larval molt (Raizen et al., 2008). RIS interneuron-defective *aptf-1(loss-of-function)* mutants have been found to be impaired for movement quiescence during DTS based on tracking the speed of the worm’s nose tip (Turek et al., 2013). To determine whether this nose movement reflects locomotor activity, we quantified the locomotor rate of these animals, while blinded to genotype, by counting the number of sinusoidal body bends per minute. We compared lethargus (A) and post-lethargus (B) movement of two *aptf-1(lf)* strains to wild type (N2) and to lethargus-defective *npr-1(lf)* animals (Choi et al., 2013). We found that unlike *npr-1(lf),* which showed a high frequency of body bends during and outside of lethargus, *aptf-1(lf)* mutants greatly reduced their frequency of body bends during lethargus to a level similar to that of N2 controls. However, we noted that these RIS-defective animals were strikingly defective in head movement quiescence and also showed rocking movements: alternating backward and forward body movements, each resulting in less than a half-body translation of the worm’s position and virtually no net movement in either direction.

In addition to developmental sleep, *C. elegans* engages in sleep following exposure to conditions that cause cellular stress (Hill et al., 2014; Nelson et al., 2014; DeBardeleben et al., 2017). This stress-induced sleep (SIS) is dependent on the peptidergic ALA interneuron and the action of several neuropeptides that collectively promote a state of coordinated behavioral quiescence (Nath et al., 2016). Because the RIS neuron has recently been implicated in regulating SIS based on frame subtraction methods (Grubbs et al., 2019; Konietzka et al., 2019), we examined the behavior of *aptf-1(lf)* animals during SIS triggered by ultraviolet light (C) and by ingestion of pore-forming Cry5B toxin (D). We compared the behavior of *aptf-1(lf)* mutants to N2 and to SIS-defective *ceh-17(lf)* animals that are impaired for ALA neuron function (Van Buskirk and Sternberg, 2010). In contrast to ALA-defective animals, *aptf-1(lf)* mutants showed wild type body-bend quiescence during SIS. As in lethargus, RIS-defective animals showed head movement, but they did not rock back and forth as they did during lethargus. In an independent assay, we examined each animal (again, blinded to genotype) for five seconds every 10-15 min following exposure to a SIS trigger and categorized their movement during that time as “fully immobile”, “body immobile but head mobile”, where head mobility was defined as any discernible movement, or “head and body mobile”, where body mobility was defined as a translation of the body position by at least 1/10 body length. Most *aptf-1(lf)* animals moved only their heads and did not translate their body position (F and J); SIS body movement quiescence in *aptf-1(lf)* was similar to wild type, but took longer to set in in the case of UV-SIS (G). These data indicate that the RIS neuron plays a major role in controlling head movement quiescence, with a relatively minor impact on body movement quiescence, at least when examined on standard NGM plates. It will be of interest to determine whether the impact on body movement quiescence is a secondary consequence of head movement. Though head movement is often referred to as foraging behavior, we did not observe any feeding (pharyngeal pumping) in *aptf-1* mutants in these assays. Our results have implications regarding the circuits regulated (head-only versus head and body) by RIS and ALA activity. This analysis also emphasizes the importance of visual inspection, scored blind to genotype, as a valuable tool in studies of quiescence.

## Reagents

Strains available from the CGC: N2 wild type, *IB16 ceh-17(np1),* DA609 *npr-1(ad609),* HBR227 *aptf-1(gk794),* HBR232 *aptf-1(tm3287).* Cry5B-expressing bacteria, received from Raffi Aroian, is available from our lab.
