# Erratum: *aptf-1* mutants are primarily defective in head movement quiescence during *C. elegans* sleep

**DOI:** 10.17912/micropub.biology.000162

**Published:** 2019-08-23

## Description

Robinson, B; Goetting, DL; Cisneros Desir, J; Van Buskirk, C (2019). *aptf-1* mutants are primarily defective in head movement quiescence during *C. elegans* sleep. microPublication Biology. 10.17912/micropub.biology.000148 published online Aug 19, 2019 @ 09:06; corrected after publication Aug 22, 2019.

In the version of the microPublication originally published online, the names of the third and last author were incorrect. The correct names are **Janine Cisneros Desir** and **Cheryl Van Buskirk**. These mistakes have been corrected in the html and PDF versions of the microPublication.

